# An Uncertainty Model for Strain Gages Using Monte Carlo Methodology

**DOI:** 10.3390/s23218965

**Published:** 2023-11-03

**Authors:** Matthias Haslbeck, Jörg Böttcher, Thomas Braml

**Affiliations:** Universität der Bundeswehr München, Werner-Heisenberg-Weg 39, D-85577 Neubiberg, Germany; joerg.boettcher@unibw.de (J.B.); thomas.braml@unibw.de (T.B.)

**Keywords:** measurement uncertainty, electric strain measurement, GUM, global sensitivity analysis, Monte Carlo simulation, model updating

## Abstract

For the purpose of validation and identification of mechanical systems, measurements are indispensable. However, they require knowledge of the inherent uncertainty to provide valid information. This paper describes a method on how to evaluate uncertainties in strain measurement using electric strain gages for practical engineering applications. Therefore, a basic model of the measurement is deduced that comprises the main influence factors and their uncertainties. This is performed using the example of a project dealing with strain measurement on the concrete surface of a large-span road bridge under static loading. Special attention is given to the statistical modeling of the inputs, the underlying physical relationship, and the incorporation and the impact of nonlinearities for different environmental conditions and strain levels. In this regard, also experiments were conducted to quantify the influence of misalignment of the gages. The methodological approach used is Monte Carlo simulation. A subsequent variance-based sensitivity analysis reveals the degree of nonlinearity in the relationship and the importance of the different factors to the resulting probability distribution. The developed scheme requires a minimum of expert knowledge of the analytical derivation of measurement uncertainties and can easily be modified for differing requirements and purposes.

## 1. Introduction

### 1.1. Motivation

The application of structural health monitoring concepts, methods of system identification, or other data-driven assessment tools mandatorily requires the evaluation of measurement uncertainties in order to provide valid information [1].

The necessity to provide engineering models for the uncertainty of strain gages has already been highlighted in many publications, such as [2,3,4,5,6], where specially tailored models are presented for special applications. However, a fundamental article about the special scope of uncertainty evaluation in strain measurements on large structures could not be identified, but is urgently needed for the data-driven assessment of structures [7,8].

Especially in outdoor applications under unsteady environmental conditions, a feasible way for propagating the uncertainties inherent to the gained data is necessary [9]. Certain parameters often can only be estimated roughly, and are neglected due to difficulties in modeling the analytical relations, or the output does not meet the requirement of a Gaussian distribution function to model the deviation from the “true value”. Additionally, the derivation of the analytical expression from a complex measurement process, nonlinear measurement models, and the formation of (higher-order) partial derivatives cause problems, making classical approaches such as the Gaussian law of propagation of uncertainties as described in [1] often infeasible [10]. Therefore, the Monte Carlo methodology can help to provide valid information on uncertainties in strain measurement as previous work shows, published, e.g., in [11].

Consequently, a hands-on evaluation method for the propagation of uncertainty of electric strain measurement is needed that incorporates the major influences on the test results, but is still applicable in engineering practice. More theoretical approaches like in [3,12,13,14] are not well tailored to the use cases in civil engineering and give little practical advice.

### 1.2. Scope of This Paper

In order to provide a statistical model of uncertainty for electric strain gages mounted on a measuring object made of concrete, the main influence factors on the uncertainty in the data are given in this contribution. Therefore, the most influential parameters from the measuring devices themselves, the interaction between the concrete structure and the strain gage, and human error in the process of mounting are explained and quantified.

The underlying literature study and the analysis of manufacturing data are supplemented by an experimental study on the error from misalignment of the strain gage.

To capture all relevant parameters, a probabilistic model of the measurement is derived from the physical relationship of the indirect measurement of strain using differences in electrical resistance. Using the Monte Carlo methodology in combination with methods for variance reduction in the sampling process, the standard uncertainty and the type of distribution function are presented for the example of measured data from load testing conducted at a concrete bridge.

After a thorough discussion of the resulting distribution, a global sensitivity analysis is performed. Due to the high nonlinearity of the model, the sensitivity study is implemented using Sobol sensitivity indices. The discussion of the results reveals starting points for increasing the preciseness of strain measurement in civil engineering applications. The developed model and its implementation can easily be adapted to all fields of engineering where electric strain gages are used and thus have high significance. As the method of Monte Carlo simulation remains applicable for all kinds of input–output relations and requires only a minimum of expert knowledge on the analytical derivation of measurement uncertainties, the proposed procedure is easily applicable for heteroscedastic measurement results and diverging types of distribution functions.

### 1.3. Measurements and Applied Equipment

**Figure 1:**
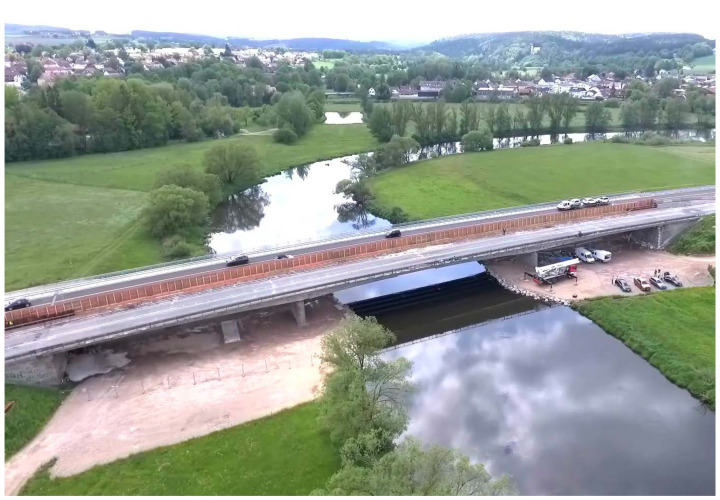
Overview screen of the test bridge in Roding from [21].

To give an illustration on the proposed model, data from a measuring project conducted from 23 to 24 May 2019, with the objective of gaining data for a subsequent identification of system parameters tailored to a refined structural analysis of a three-span box girder bridge is used. The bridge with a total length of 137 m was built in 1965 from concrete of strength class B300 according to DIN 1045:1959-11 with common aggregates. An overview of the structure is given in Figure 1. Additional information on the project can be found in [15,16,17].

**Figure 2:**
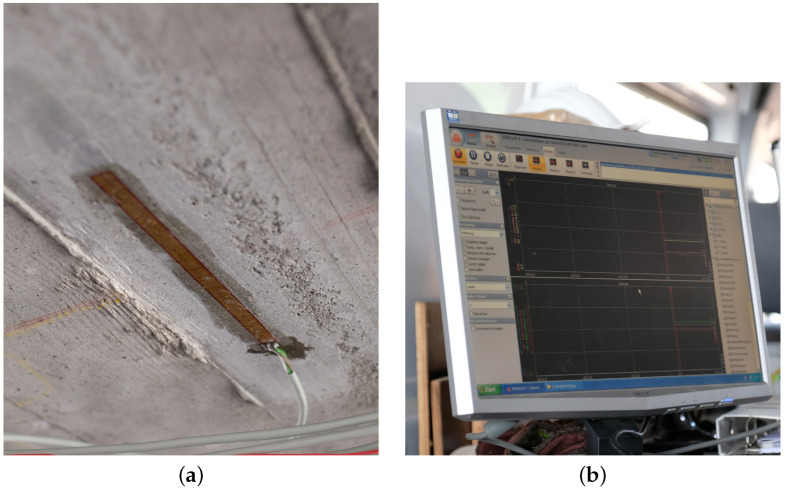
(**a**) Strain gage mounted on the superstructure; (**b**) test results visualized in the data acquisition system.

At the bottom side of the superstructure, strain gages were installed as quarter bridges in a three-wire configuration. The applied product is specially adapted for the application on concrete as its grid length of 120 mm helps to avoid local effects from material inhomogeneity and to average the strain at a larger zone [18]. Due to the high grid length, transverse sensitivity can be neglected. A picture of the strain gage installed on the superstructure is shown in Figure 2a.

The strain gages of type KC-120-120-A1-11 produced by KYOWA Co., Ltd., (Hiroshima, Japan) fulfill the quality standard of VDI2635-1 [19] and OIML R 62 [20]. The applied product is especially tailored to the application on concrete surfaces concerning their thermal expansion. As an adhesive, an acrylate with the product code CC-35x5 produced by the same manufacturer has been applied to meet the requirements of the installation guidelines for the used strain gages. As a data acquisition system, a DS-NET BR8 has been used, which is distributed by ZSE Electronic GmbH (Bietigheim-Bissingen, Germany). This component comprises the Wheatstone bridge circuit, the measurement amplifier, and the analog–digital converter. At the time of measurement, all three devices had a current calibration certificate to show conformity to the manufacturer’s specifications on the measurement uncertainties. The visual output from the data acquisition system is given in Figure 2b.

**Figure 3:**
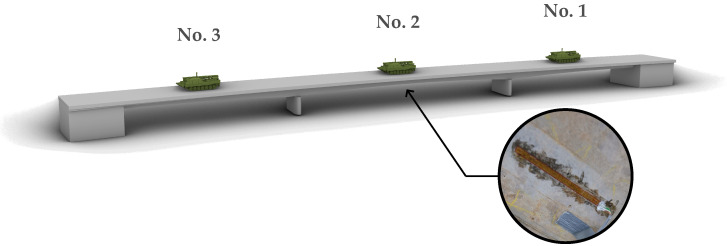
Illustration of the measurement project concerning measurement location and load position no. 1, 2 and 3.

The measured strains were caused by a recovery tank placed in the middle of each span as static load. Figure 3 gives a view of the bridge from below and illustrates the applied measurement devices and their position as well as the static loading on position nos. 1–3.

Special challenges for this type of bridge surveillance are the application of the sensor systems from a mobile elevating work platform and the unsteady environmental conditions from which additional uncertainties of the measured values arise.

## 2. State of the Art

### 2.1. Foil-Type Strain Gages for Measurement on Concrete

**Figure 4:**
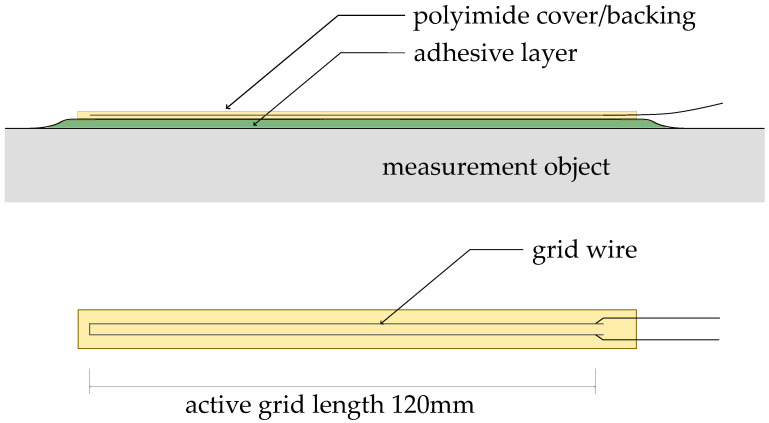
Components of foil-type strain gage, after [18].

Subsequent to the development of foil-type strain gages in the 1950s, foil-type strain gages have become the standard sensor in the electric strain measurement [18]. The mechanical changes of strain on the concrete surface are transferred to the included electrical conductor, changing its electrical resistance according to its alteration of length [18]. The foil-type strain gage itself is composed of a backing from polyimide, a constantan wire-grid, and a thin cover [18]. A schematic description of the different constituent parts of a foil-type strain gage is given in Figure 4. For the application on concrete surfaces, grid lengths of at least 120 mm and the implementation of only two conductors in the foil have proven to be a convenient choice [18]. However, the application on large structures requires the application of quarter bridges and leaves no chance for calibrating the measurement chain on-site.

The process of application on concrete objects requires the filling of inevitably present pores, the smoothening of the surface, grinding, and the application of an adhesive to ensure a loss-free transfer of the strain from the object to the strain gage [18]. Within this contribution, the bonding is supposed to be error-free. For a closer insight into the complex mechanical model of strain transfer and the model introduced by Hönisch, please refer to [22,23,24]. Effects of creeping in the strain gage can be neglected due to the low number of strain cycles.

### 2.2. Evaluation of Measurement Uncertainties According to GUM

#### 2.2.1. Analytical Approximation

As a standard document for the evaluation of uncertainties, the “Guide to the Expression of Uncertainty in Measurement” (GUM) was introduced in 1993 by the “Joint Committee for Guides in Metrology” and gives suggestions on the analytical derivation of measurement uncertainties. For the technical terms in this section and a more detailed explanation of the subject, please refer to [1].

The core of the proposed methodology in [1] is referred to as the general law of error propagation using statistical methods. Different from the concepts of the maximum permissible error, the derived uncertainty measure represents the distribution of possible measurement reading according to the probability of occurrence when repeating the experiment a large number of times. The evaluation of a combined standard uncertainty $u_{c}$ of N independent input quantities in Equation (1) is based on the Gaussian error propagation method, which includes a quadratic superposition of the different components, which implies equal treatment of systematic and random components. For the weighing of the different components $u(x_{i})$ for the physical relation utilized in an indirect measurement method expressed by a function *f*, the partial derivatives of first order $\frac{\partial f}{\partial x_{i}}$ are applied, which can be interpreted as sensitivity coefficients [1]. Equation (1) is a first-order approximation, so it can be misleading in case of nonlinear functional relations. However, this is the case for electric strain measurements. Additionally, GUM assumes normally distributed measurement deviations, which are not necessarily the case for electric strain measurement as we will be shown in Section 5.

Even though the approach of GUM [1] gives a simple and straightforward way of treating uncertainty components, the computation of partial derivatives, e.g., by finite differences, the implementation for complex input–output relations can be demanding [10], and the verification of the underlying assumptions is in most cases not feasible.

(1) $$u_{c}=\sqrt{\sum_{i=1}^{N}{\left(\frac{\partial f}{\partial x_{i}}\cdot u\left(x_{i}\right)\right)}^{2}}$$

#### 2.2.2. Monte Carlo Methodology and the GUM Uncertainty Framework

For a more intuitive and less parametric way of evaluation, Supplement 1 of GUM [10] gives guidance to evaluate the uncertainties inherent to measurement results.

In a Monte Carlo simulation, complex systems are analyzed by using a randomized simulation of a large number of input samples and observing the distribution of the output [25]. The theoretical justification for this approach is the law of large numbers, which guarantees that the conclusions drawn from the limited number of simulation runs correspond to the true results if only the number of runs is large enough [26]. In order to limit the necessary number of runs, methods of variance reduction are advisable. Therefore, the sampling of the input in this contribution is performed using the Latin Hypercube sampling strategy, which ensures a representative spreading of the samples on the parameter space [27]. A drawback of the method is that the number of runs needs to be specified before starting the evaluation. However, the simulation time is commonly short enough to either use a number of samples that is surely sufficient to reach the required preciseness or to iteratively increase the number of runs. Theoretical background of the sampling procedure in a Latin hypercube is given in [28,29,30].

In order to specify the accuracy of the test results, the methodology of bootstrapping is applied. Bootstrapping is a way of quantifying the accuracy inherent to empirical distribution functions. The algorithm resamples with replacement from the test results and judges on the accuracy of the estimators by comparing the computed values of the measure under study [31]. Different from other methods, this approach is applicable to any test data and does not rely on too restrictive assumptions concerning the parent distribution of the test data [32]. In this contribution, bias-corrected and accelerated bootstrap confidence intervals are applied to determine the accuracy of the results and to decide on the applicable measure of dispersion. As a thorough explanation of this methodology is not feasible due to brevity and not within the scope of this journal, it is referred to [33,34,35] for further explanation, and for the application for empirical uncertainty estimation, please refer to [21].

**Figure 5:**
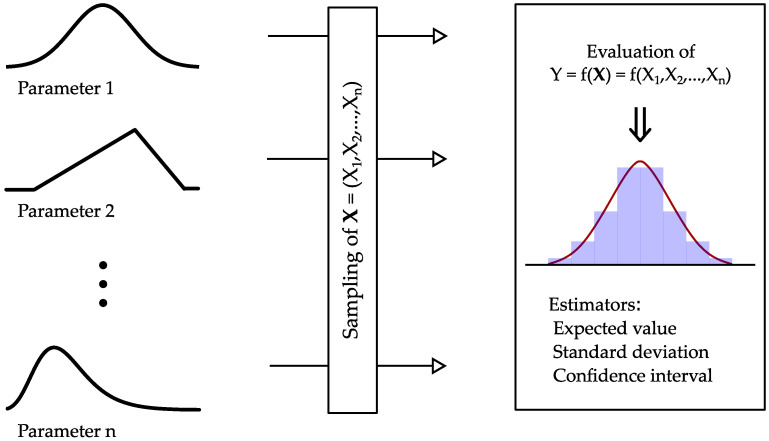
Illustration of the procedure proposed in [10] to evaluate the distribution and the dispersion of the possible results.

Figure 5 gives an illustration of the basic methodology of uncertainty propagation using the Monte Carlo methodology. After the process of sampling an input vector X from the different components of uncertainty, the physical relation is exploited to simulate the indirect measurement for varying inputs. The obtained output sample is then evaluated according to its measures of central tendency and dispersion (mean value and standard deviation). From a more metrological point of few, GUM [10] describes the procedure as follows:

1. **Formulation**: Determination of the input by its probability function and modeling the functional relation of input and output variables of the indirect measurement procedure;
2. **Propagation**: Starting the Monte Carlo runs until a sufficient number of runs are obtained to ensure convergence;
3. **Summarizing**: Evaluation of the resulting vector of simulation results concerning measures of central tendency and dispersion.

This procedure, called GUM uncertainty framework, is applicable to hardly all indirect measurements, but requires a thorough knowledge on the distribution of the input variables and the physical effect exploited for the determination of the measurand and to build up the measuring model [9].

Besides its enormous potential for the propagation of uncertainty, the Monte Carlo methodology can also be applied to gain global measures of sensitivity as described in Section 2.3 of this paper. This study is advisable to get insight into the main influence factors for the uncertainty inherent to the measurement results.

### 2.3. Variance-Based Sensitivity Analysis

Methods that are capable of assigning the contribution of each influence factor to the total uncertainty of a certain result are referred to as sensitivity analysis tools [36,37,38,39]. In the context of uncertainty quantification of measurement results, the apportioning of sensitivity measures to an uncertain input can help to prioritize input variables in further investigations in order to decrease the combined uncertainty in the measurand. Depending on the complexity and the mathematical structure of the investigated model function, different methods are appropriate and have their own strengths and weaknesses [37].

Due to reasons of brevity, the different methods cannot be described in detail here. Instead, readers are referred to [37] for a detailed explanation of the different approaches and to [39,40] for an overview and decision-making aid on the application of different measures of sensitivity.

The available measures can be characterized by their validity in the feasible parameter space. Local measures, mainly built up on partial derivatives at a fixed nominal value, are strictly speaking only valid at a certain basic point. The concept of local sensitivity coefficient is anchored to [1] as it is part of the determination of combined uncertainties using the Gaussian error approximation. However, the application of this scheme disregards the interaction between input quantities when nonadditive, nonlinear, or nonmonotonous models are applied in the process of uncertainty propagation. Therefore, this contribution argues in favor of global methods that are model-free, i.e., independent from the mathematical formulation of the model function [37,41], truly global in the sense that they remain valid for the entire range of the parameter space [42], and easy to interpret by practitioners [43]. In this case, however, the prerequisites for a simple evaluation using partial derivatives are not given due to the nonlinear and nonadditive nature of the output function, which necessitates advanced methods. Of special interest in the field of metrology using the Monte Carlo methodology is the application of Sobol indices, which are explained in this section. The required measure of sensitivity relies on a procedure that is easy to implement using again a Monte Carlo scheme. Due to the automatic generation of sensitivity measures regardless of the specific setting, it has high relevance for practical application in the evaluation of measurement uncertainty.

Variance-based methods such as the one presented by Ilya M. Sobol in [44] split the overall variance V=V(f(X)) of the output into the shares that can be attributed to each input factor $X_{i}$, abbreviated by $V_{i}$, and terms for interactions for i,j,…n expressed by the terms $V_{ij}$ to $V_{1,2,\ldots ,n}$ as shown in Equation (2).

(2) $$V=V\left(f\left(X\right)\right)=\sum_{i=1}^{n}V_{i}+\sum_{1\leq i<j\leq n}^{}V_{ij}+\cdots +V_{1,2,\ldots ,n}$$

From the decomposed variance in Equation (2), the respective sensitivity indices after Sobol can be derived according to Equation (3) for the main effect of each input variable $S_{i}$ as well as for the interaction of the arbitrary order $S_{ij},S_{ijk},\ldots$. Using the definition from (3), the decomposition from Equation (2) can be reformulated as shown in Equation (4). It should be noticed that, here, the sum over all indices sums up to one.

(3) $$S_{i_{1},\ldots ,i_{s1}}=\frac{V_{i_{1},\ldots ,i_{s1}}}{V}$$

(4) $$1=\sum_{i=1}^{n}S_{i}+\sum_{1\leq i<j\leq n}^{}S_{ij}+\cdots +S_{1,2,\ldots ,n}$$

On the basis of these fundamental definitions, both the first-order effect index and the total effect index can be distinguished. Equation 5 shows the definition of the first-order sensitivity indices $S_{i}$. The total effect indices $S_{Ti}$ comprise all terms of Equation (4) containing a certain factor $X_{i}$. For convenience, total effect indices are commonly computed from the difference of the sum of those variances independent from *i*, abbreviated by $V_{∼i}$, to the theoretical sum of all sensitivity indices from Equation (4), i.e., one. The respective relation is given in Equation (6). For model functions of a nonadditive structure, the sum of first-order sensitivity indices $\sum_{i=1}^{n}S_{i}$ is not equal to one. The residue can thus be interpreted as a measure for nonadditive parts in the model. Additionally, the comparison of $S_{i}$ and $S_{Ti}$ can give information on the impact of nonadditive parts of the model function for each influence factor.

(5) $$S_{i}=\frac{V_{i}}{V}$$

(6) $$S_{Ti}=1-\frac{V_{∼i}}{V}$$

Except for some probability functions that can easily be computed manually, the required variances are commonly computed using the Monte Carlo method. The approach proposed by Andrea Saltelli in [45] and described hereafter is easy to implement and is valid under very mild preconditions.

Using a random sampler, two matrices, A and B, are formed where each row can be interpreted as input for one Monte Carlo run and the number of columns is the sample size to ensure convergence of the simulation. In most cases, it is advisable to employ variance reduction techniques, such as Sobol sequences, to reduce computational cost. A representation of the resulting matrices A and B can be seen in Expression (7), where A and B are the first and the second part of matrix columns from a pseudo random sample with *N* rows and twice the number of inputs as columns. These matrices A and B are then transformed according to the respective distribution function of the inputs. For further information on the numeric calculation procedure, please refer to [46] for the algorithm and to [27,47] for the sampling strategy used to generate Sobol sequences.

(7) $$\begin{array}{c} A=\left[\begin{array}{ccccc} a_{11} & \cdots & a_{1i} & \cdots & a_{1n}\\ \vdots & \; & \vdots & \; & \vdots \\ a_{21} & \cdots & a_{2i} & \cdots & a_{2n}\\ \vdots & \; & \vdots & \; & \vdots \\ a_{N1} & \cdots & a_{Ni} & \cdots & a_{Nn} \end{array}\right]\Rightarrow y\left(A\right)=\left[\begin{array}{c} y(A_{1})\\ \vdots \\ y(A_{2})\\ \vdots \\ y(A_{N}) \end{array}\right]\\ B=\left[\begin{array}{ccccc} b_{11} & \cdots & b_{1i} & \cdots & b_{1n}\\ \vdots & \; & \vdots & \; & \vdots \\ b_{21} & \cdots & b_{2i} & \cdots & b_{2n}\\ \vdots & \; & \vdots & \; & \vdots \\ b_{N1} & \cdots & b_{Ni} & \cdots & b_{Nn} \end{array}\right]\Rightarrow y\left(B\right)=\left[\begin{array}{c} y(B_{1})\\ \vdots \\ y(B_{2})\\ \vdots \\ y(B_{N}) \end{array}\right] \end{array}$$

For each of the analyzed input factors $X_{i}$, another matrix $A_{B}^{\left(i\right)}$ is derived, where the $i^{th}$ column of A is replaced by its counterpart in B as shown in Expression (8).

(8) $$A_{B}^{\left(i\right)}=\left[\begin{array}{ccccc} a_{11} & \cdots & b_{1i} & \cdots & a_{1n}\\ \vdots & \; & \vdots & \; & \vdots \\ a_{21} & \cdots & b_{2i} & \cdots & a_{2n}\\ \vdots & \; & \vdots & \; & \vdots \\ a_{N1} & \cdots & b_{Ni} & \cdots & a_{Nn} \end{array}\right]=>y\left(A_{B}^{\left(i\right)}\right)=\left[\begin{array}{c} y(A_{B_{1}}^{\left(i\right)})\\ \vdots \\ y(A_{B_{2}}^{\left(i\right)})\\ \vdots \\ y(A_{B_{N}}^{\left(i\right)}) \end{array}\right]$$

After the sampling of A, B, and the derived samples $A_{B}^{\left(i\right)}$, the model function, in this case, the evaluation of the corresponding strain is computed. The respective vectors are denominated by $y\left(A\right),y\left(B\right),\;\mathrm{and}\;y\left(A_{B}^{\left(i\right)}\right)$.

For the determination of the sensitivity indices $S_{i}$ and $S_{Ti}$ for each input variable $X_{i}$, several estimators have been developed in the last decades. In Equations (9) and (10), the computation rules for the estimators introduced for the first time in [48] and explained thoroughly, e.g., in [43,49,50], are given, which are among the most efficient ones and therefore applied in this paper.

As the basis of every kind of sensitivity analysis is the choice of the evaluated parameters and the uncertainty associated with the different influences, the assumed distribution functions and their relationship to the measurement reading are elaborated in the following section.

(9) $$S_{i}=1-\frac{\frac{1}{2N}\sum_{v=1}^{N}{\left(f{\left(B\right)}_{v}-f{\left(A_{B}^{\left(i\right)}\right)}_{v}\right)}^{2}}{\frac{1}{N}\sum_{v=1}^{N}{\left(f{\left(A\right)}_{v}-\frac{1}{N}\sum_{v=1}^{N}f{\left(A\right)}_{v}\right)}^{2}}$$

(10) $$S_{Ti}=\frac{\frac{1}{2N}\sum_{v=1}^{N}{\left(f{\left(A\right)}_{v}-f{\left(A_{B}^{\left(i\right)}\right)}_{v}\right)}^{2}}{\frac{1}{N}\sum_{v=1}^{N}{\left(f{\left(A\right)}_{v}-\frac{1}{N}\sum_{v=1}^{N}f{\left(A\right)}_{v}\right)}^{2}}$$

## 3. Considered Measurement Uncertainties

### 3.1. Overview

**Figure 6:**
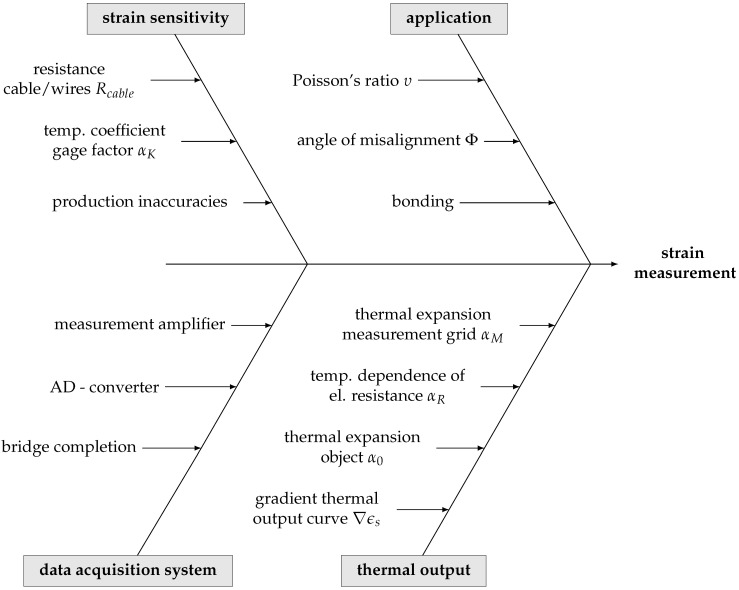
Ishikawa diagram of the potential causes of uncertainty in the measurement chain.

In order to identify and visualize potentially significant sources of uncertainty, the components of the measurement chain need to be reviewed. The Ishikawa diagram in Figure 6 shows the four main contributions to the output estimate that are examined further in the latter.

The transfer of the difference in voltage to an attributed strain value, as part of the data acquisition system, is studied as well as the different influences of temperature on the measurement reading in the absence of mechanical strain, e.g., due to scatter in the thermal characteristics of the measurement object and the sensor. Contributions from misalignment are examined in Section 3.3 using an experimental study for the specific use case. Error arising from the adhesive layer between the concrete surface and the strain gage is not considered in the context of this paper. Instead, it is quantified as a blunder. Measurement readings biased by bonding failure may be detected by an outlier test and discarded or respected in a different manner.

The assumed strain sensitivity of the gage is examined using the functional relationship of the measurement principle to perform the quantification of the combined uncertainty in Section 5. Influences from production inaccuracy and temperature are discussed using literature references and the manufacturer’s specifications.

The different categories of influence factors and the attributed sub-factors are considered mutually independent, which is in accordance to [1].

### 3.2. Strain Sensitivity (k-Factor)

Due to the implicit derivation of the mechanical strain from changes in resistance of a wire, a transmission factor (gage factor) is applied to assign the difference in electric resistance of the gage to the change in strain of the measured object. The sensitivity of the strain gage (k-factor) is defined as the ratio of the change in electric resistance $\frac{dR}{R}$ and a priori known strain value $\epsilon_{0}$ [20]. As a direct measurement of resistance is not feasible, Equation (11) also contains the relation for the difference in voltage ΔU compared with the excitation voltage $U_{0}$ using a bridge factor of n=4 due to the design of the Wheatstone bridge circuit. As the observed strain values in bridge engineering are generally small and still in the elastic range, the sensitivity of the examined gage is assumed to be constant [18] and the characteristic of the applied Wheatstone bridge circuit is linear [51]. The requirements of the testing procedure to determine $k_{0}$ are described in [19,20]. As the evaluation of the strain factor is part of the ongoing sampling inspection [19], the uncertainty of the gage factor is given in the strain gage package information.

(11) $$\frac{dR}{R}=k_{o}\cdot \epsilon_{0}\approx \frac{\Delta U}{n\cdot U_{0}}$$

Due to imperfect manufacturing, the sensitivity of the single gages can diverge. According to the manufacturer‘s specifications, the gage factor under standard climate conditions (T = 24 °C) can be expected to be $k_{0}=2.13\;\pm \;1.0\%$. The given uncertainty in the specification of the gage factor due to manufacturing is assumed to be an expanded measurement uncertainty for a 95% quantile.

Thus, a standard deviation of $1.09\times 10^{-2}$ is derived for $k_{0}$. According to [19], the basic population is assumed to be normally distributed. From this follows an input variable $k_{0}$ with $X_{k_{0}}∼N\left(2.13;1.9\times 10^{-2}\right)$.

As the cases of application normally do not meet the test specification in [20] and due to the sensitivity of the gage factor to changes in temperature, an additional component of uncertainty needs to be respected. The addition in sensitivity is linear to the difference in temperature ΔT and can be expressed according to Equation (12), which has been taken from [18].

(12) $$k_{T}=k_{0}\cdot \left(1+\alpha_{K}\cdot \Delta T\right)$$

The temperature coefficient gage of the factor can be expected to be $\alpha_{K}=1.5\times 10^{-4}\frac{1}{K}$. According to the manufacturer’s specifications, $\alpha_{K}$ is assumed to be

$\alpha_{K}=1.5\times 10^{-4}\frac{1}{K}\pm 5.00\times 10^{-4}\frac{1}{K}$, and its possible values are uniformly distributed. Consequently, the random variable can be expressed by $X_{\alpha_{K}}∼U\left(1.0\times 10^{-4}\frac{1}{K};2.0\times 10^{-4}\frac{1}{K}\right)$. The difference in temperature ΔT is to be interpreted as the difference of the measurement object from the reference temperature. The reference in this case is 24 °C according to the package information.

As an additional influence, the loss of sensitivity due to the ohmic resistance of the cable wire $R_{cable}$, relative to the resistance of the strain gage $R_{gage}$, needs to be taken into account according to Equation (13). This effect, however, is compensated precisely by the measurement amplifier and can thus be neglected for the presented measuring setup.

(13) $$k^{★}=k_{T}\cdot \frac{R_{gage}}{R_{gage}+R_{cable}}$$

### 3.3. Inaccuracies in the Application Process

#### 3.3.1. Theoretical Background

Due to its buildup, a single electrical strain gage is a one-dimensional sensor, so strain is only measured in one direction. This is especially true for the used type of 120 mm length, where transverse sensitivity is zero in good approximation. One possible source of uncertainty is the misalignment of the strain gage. Misalignment in the context of this paper means that the direction in which the strain gage is positioned does not coincide with the desired direction of strain evaluation. This results in a distorted measurement result.

In case of misalignment, the recording consists of one share from the targeted direction and another from the transverse direction. For the specific use case of measuring strain below the girders, the stress state on the measuring object can be assumed to be uniaxial, so the transverse strain is linearly coupled to the principal strain $\epsilon_{1}$ via Poisson’s ratio ν of the concrete surface [18].

**Figure 7:**
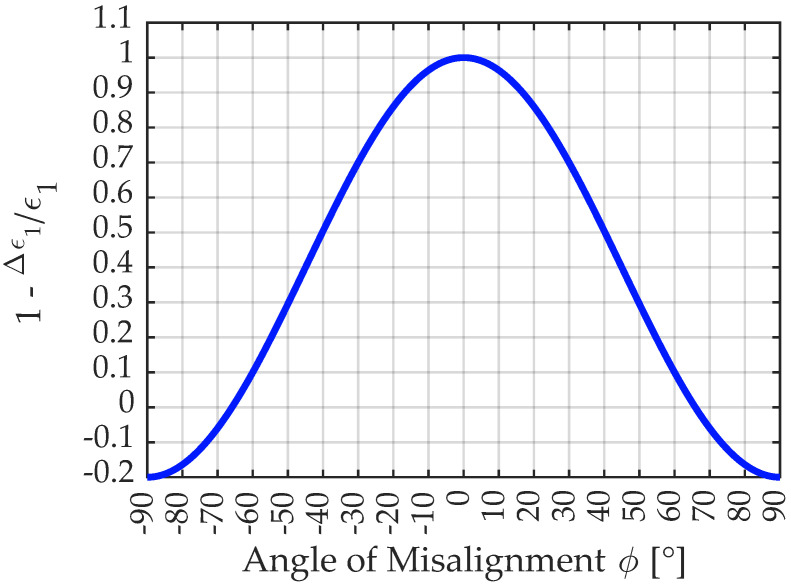
Illustration of the decline in measurement recording for different angles of misalignment for a Poisson’s ratio of ν=0.2.

As transverse sensitivity is zero and the stress state is uniaxial, the recording of strain is always smaller than the actual value when the gage is misaligned, which can be interpreted as a decline of the k-factor according to Equation (14) from [18]. Figure 7 shows the ratio between the measured value $\epsilon_{1}-\Delta \epsilon_{1}$ and the actual strain $\epsilon_{1}$ depending on the angle of misalignment as it is also used in Equation (14). As the graph is symmetric to Φ=0, each misalignment of the strain gage, no matter what the direction of the misalignment is, leads to a decrease in the evaluated strain and thus biased results. For the necessity on the correction of this systematic uncertainty component, please refer to Section 3.3.3.

(14) $$\tilde{k}=k_{T}\cdot \left(1-\frac{\Delta \epsilon_{1}}{\epsilon_{1}}\right)=k_{T}\cdot \frac{\left(1-\nu )+(1+\nu )\cdot \cos(2\Phi \right)}{2}$$

#### 3.3.2. Experimental Study and Results

**Figure 8:**
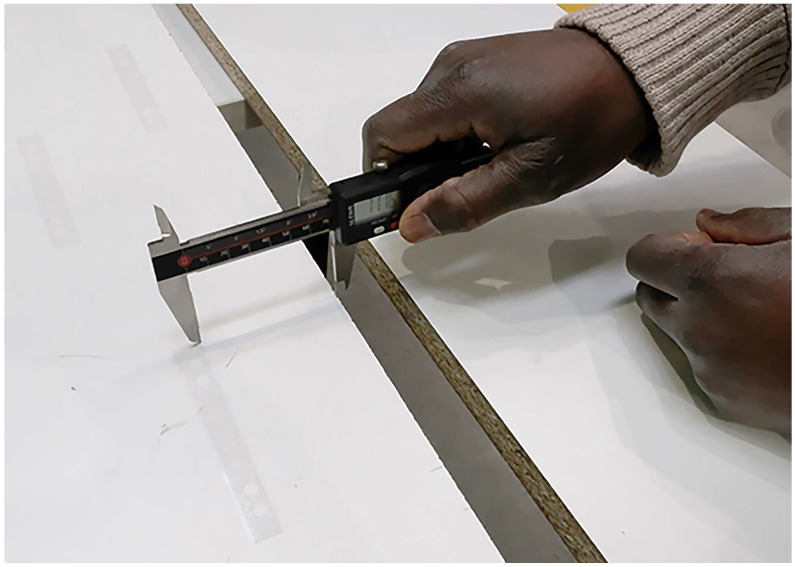
Evaluation of the angle of misalignment using a sliding caliber.

**Figure 9:**
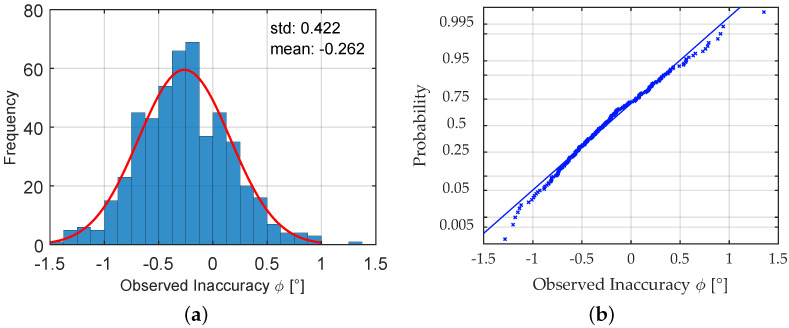
(**a**) Histogram of the evaluated angle of misalignment from a sample of size 500; (**b**) normal probability plot of the observed inaccuracy from misalignment.

In order to estimate the expected inaccuracies from a possible misalignment, an experimental study was conducted. For the evaluations, a number of 500 dummies of strain gages were used that were applied to boards that resemble the form boards used when the experimental bridge was built. Form boards are wooden boards used as a form to pour the concrete in. They lie close to each other and follow the direction of the structure. Thus, they give a similarly helpful aid for orientation compared with the imprints of the form board that could be found on-site. The gages were installed by an engineer with a certain experience in the mounting of electric strain gages in industrial applications. Thus, his work reflects the accuracy that can be expected for this use case. For the determination of the angle of misalignment, a precise sliding caliber was used. An impression of the procedure of evaluating the angle of misalignment is given in Figure 8. Figure 9a shows a histogram of the angles of misalignment computed from the results and a normal fit to the results, while Figure 9b visualizes the similarity to the Gaussian distribution in a normal probability plot. The empirical distribution function can be characterized by its expected value of μ≈0.262° compared with the axis and standard deviation σ≈0.442°. The 95% confidence intervals for the two estimators can be given by [0.225;0.299] for μ and [0.396;0.453] for σ and were evaluated using the bootstrap methodology.

#### 3.3.3. Assumed Random Variables

For the evaluation of uncertainties in the installation process, the angle of misalignment is modeled by a random variable $X_{\Phi }∼N\left(0.262;0.422\right)$. As Poisson’s ratio does very much depend on the (in most cases unknown) kind of concrete and its production, the entire range of possible values according to [52] shall be applied, stochastically modeled as uniformly distributed according to $X_{\nu }∼U\left(0.14;0.26\right)$.

Due to the bias in the data (μ≠0°) and the fact that any error from misalignment leads in any case to a lower measured value of strain, the systematic error theoretically needs correction. The assigned value of this correction would be 0.0089% of the recorded value, making correction in most cases insignificant from a practical point of view.

### 3.4. Data Acquisition System

The measuring device referred to as data acquisition system is a composition of the measurement amplifier and the analog–digital converter and includes the bridge completion.

In contrast with the previously mentioned components of uncertainty, the measurement device is modeled using its maximum admissible error $\Delta y_{max}$ for a certain measurement reading $\frac{\Delta U}{U_{0}}$, here abbreviated by $MR_{x}$, as the types of the different distribution functions are hard to determine and show inherent dependencies. The related standard deviation is computed according to Equation (15), which is consistent with common assumptions for the threshold values in the manufacturer’s information. Systematic error is assumed to be zero as the device has been highly precisely calibrated. The error from analog-to-digital conversion can be quantified to be far less than the last reasonable digit of the measurement result and thus neglected. Additionally, the inserted measurement reading is a mean of several thousands of single recordings over several seconds to cover the entire range of noise components, which results in a very low empirical standard deviation of the measurement recordings of the raw data $<10^{-9}$. For this reason, noise voltage is also neglected.

As the data sheet gives unreasonably high values for the maximum assumable error from nonlinearities of the data acquisition system compared with expert experience, the maximum value from the calibration certificate is assumed.

(15) $$\sigma_{amplifier}=\left|\begin{array}{c} \frac{{\left(\frac{\Delta U}{U_{0}}\right)}_{max}}{3} \end{array}\right|$$

For the measurements, a supply voltage (SV) of 2VDC was applied. In order to quantify the influence of the board temperature, a value of $\Delta T_{board}=1K$ was assumed for every measurement. This value seems appropriate due to the relatively short time span for the test series and the self-compensation due to the internal air-cooling system. As the entire system was run in a measurement vehicle, approximately stable environmental conditions can be attributed to the measurement procedure. Table 1 gives the different components of the assumable maximum error according to the manufacturer’s specifications.

In order to model the uncertainty of the entire device accurately, the assumable maximum error $\Delta y_{max}$ can be computed according to Equation (16). The measurement reading (MR) in this equation is regarded to be deterministic.

(16) $${\left(\frac{\Delta U}{SV}\right)}_{max}=\frac{\left[0.2\frac{\mu V}{V}\cdot SV+0.05\%\cdot MR\cdot U_{0}\right]\cdot \frac{\Delta T_{board}}{10\;K}+0.2\frac{\mu V}{V}\cdot \frac{\Delta t}{24\;h}\cdot SV+5\times 10^{-4}\;V}{SV}$$

The evaluation of the maximum error has to be run twice, both for the reference state without external loading and the loaded state. For the reference state, only the nonlinearity of the calibration curve is considered as the period of measurement is fairly short (less than 1 h). For the recording in the loaded state, all terms of Equation (16) are considered. The time span from reference to recording in loaded state Δt is applied according to the test record for each specific load position. To incorporate the contribution of the data acquisition system to uncertainty in strain measurement, the respective values are concerted to strain values using $k_{0}$ and the bridge factor n=4. Due to the high number of influential factors, the deviation from the true values is assumed to be normally distributed, which follows the recommendations in [1]. As the device is highly precisely calibrated, there is no need to respect a bias in the results, so the expected value corresponds to the measurement reading. The random variables for this component are additive to the measurement recordings and can be expressed by Expressions (17) and (18).

(17) $$X_{Rec,ref}∼N\left(0\;;\frac{{\left(\frac{\Delta U}{SV}\right)}_{ref,max}}{3}\right)$$

(18) $$X_{Rec,i}∼N\left(0\;;\frac{{\left(\frac{\Delta U}{SV}\right)}_{i,max}}{3}\right)$$

### 3.5. Thermal Output

Another relevant part of the recording cannot be traced to strain as such but only to temperature effects that add to the recording in the same way as mechanical elongation would do. Change in temperature leads to drifting of the measurement signal [18] and is thus a source of uncertainty.

The change in resistance of the measuring grid material $\alpha_{R}$, the thermal expansion of the gage $\alpha_{M}$, and the thermal expansion of the measurement object material $\alpha_{B}$ all contribute to the thermal output [18]. This effect can be expressed formally by Equation (19) according to [18] for a given difference of ΔT as the difference in temperature for both recordings and where $\alpha_{M}$ is the reference coefficient from the manufacturer’s data.

(19) $${\left(\frac{\Delta R}{R}\right)}_{therm}=\alpha_{R}\cdot \Delta T+k_{T}\cdot \Delta T\cdot \left(\alpha_{B}-\alpha_{M}\right)$$

**Figure 10:**
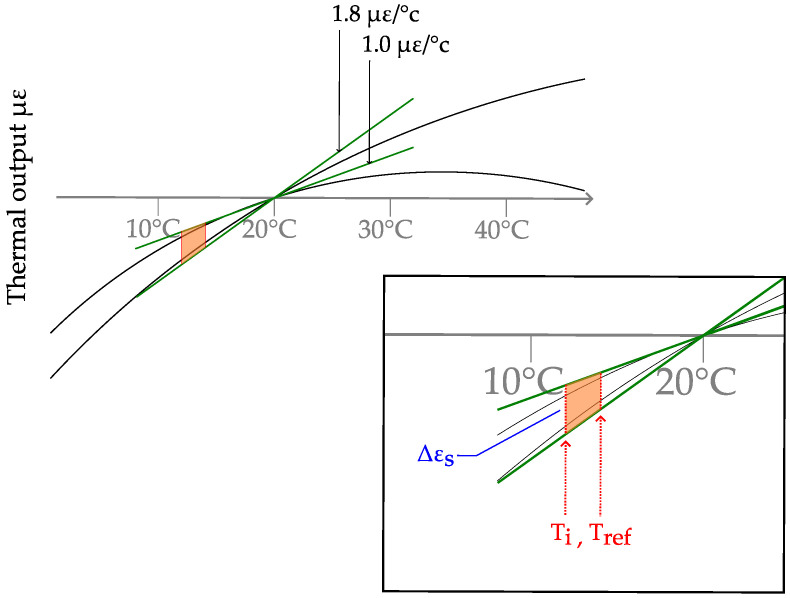
Detail of the thermal output curve and linear approximation of the minimum and maximum gradients according to the manufacturer’s specifications.

Commonly, the thermal expansion of the gage $\epsilon_{S}$ is to be specified by the manufacturer as described in [19,20] and is commonly given as a diagram. A detail of this curve is shown in Figure 10. In the range of interest, between 10 °C and 20 °C, the scatter in the thermal output is enveloped by two curves of gradient $1.0\frac{\mu m\;{}^{\circ}C}{m};1.8\frac{\mu m\;{}^{\circ}C}{m}$. It is assumed to lie between these two bounds, so the corresponding gradient of the thermal output curve $▽\epsilon_{S}$ is modeled by a uniform distribution $X_{▽\epsilon_{S}}∼U\left(1.0\frac{\mu m\;{}^{\circ}C}{m};1.8\frac{\mu m\;{}^{\circ}C}{m}\right)$. The linear approximation of the curve is given by $X_{▽\epsilon_{S}}\cdot \left(20\;{}^{\circ}C-T_{i}\right)$.

The given curve only holds when the thermal expansion coefficient of the specimen corresponds to the assumed value from the data sheet. The thermal output curve in Figure 10 is established using $\alpha_{B}\equiv 10.8\times 10^{-6}\frac{1}{K}$. As this value might be inappropriate for the specific measurement material, a correction factor needs to be introduced that is added to the computed thermal output. This correction is determined to be $\left(\alpha_{B}-10.8\times 10^{-6}\frac{1}{K}\right)\cdot \left(20\;{}^{\circ}C\right)$. The scatter of the thermal expansion of concrete from common aggregates is modeled by $X_{\alpha_{B}}∼U\left(7\times 10^{-6};13\times 10^{-6}\right)$ according to [53].

Using the beforementioned expressions, the thermal output is computed by $\epsilon_{S}=-▽\epsilon_{S}\cdot \left(20\;{}^{\circ}C-T_{i}\right)+\left(10.8\times 10^{-6}\frac{1}{K}\right)\cdot \left(20\;{}^{\circ}C-T_{i}\right)$, where the first term is the linear approximation of the curve from Figure 10, and the second is the correction for diverting values of $\alpha_{B}$.

### 3.6. Summary of Applied Probability Distribution Function

In Table 2, the implemented distribution functions for the different variables are recapitulated in standard notation.

Error from quantization in the analog–digital converter, bias from misalignment, and influence from the length of the cable wire are not regarded as they are either of inferior importance or accurately compensated by the measurement system itself.

## 4. Probabilistic Model for Uncertainties in Measurement of Concrete Constructions

**Figure 11:**
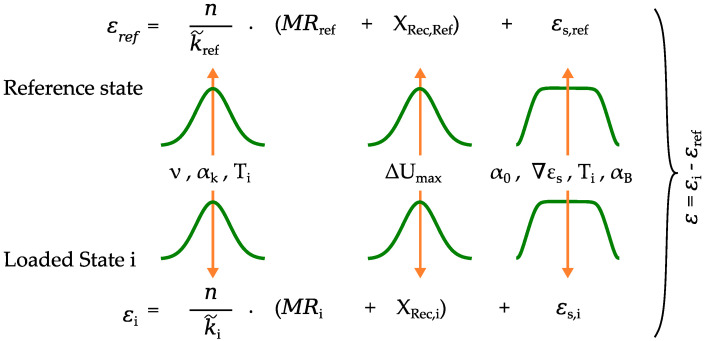
Illustration of the statistical modeling in the Monte Carlo simulation for the reference measurement and the strain evaluation at the loaded state no. i composed of sensitivity of the sensor $\tilde{k}$, measurement reading MR, error in the voltage metering $X_{rec}$, and thermal output $\epsilon_{S,X}$.

In order to describe the developed uncertainty in the measurement reading using the Monte Carlo methodology, the implementation and the simulation procedure are explained in this chapter. Figure 11 gives a visual representation of the concept.

**Input values**

The input values for the simulation consist of the measurement of voltage at both the reference as $MR_{ref}$ and the loaded state as $MR_{i}$ as well as the assumed temperature for both measurements.

Due to the high sampling frequency and the resulting high number of samples applied for averaging, the measurement reading is treated deterministically. If this assumption does not hold for the specific use case, the preciseness of the reading is modeled using the experimental standard deviation of the evaluated time series. The temperature range for each evaluation of strain is described by the minimum and the maximum values assumed during the test. The difference of the two values is referred to as ΔT in Section 3.

**Strain sensitivity**

Scatter in the strain sensitivity coefficient from production inaccuracy, application, and the shift in value due to temperature effects is superposed according to Section 3.1. If compensation for cable effects is not carried out internally by the data acquisition system, Equation (13) needs to be respected as well; however, this is not the case for the applied measurement system in the investigated example. The corresponding strain sensitivity is denoted as $\tilde{k}$. Error arising in the process of application (see Section 3.3) is incorporated only partly into the model. As blunder from imperfect bonding is infeasible to describe analytically, the consideration is restricted to effects from the misalignment of the strain gage. Therefore, scatter in the angle of alignment to the aspired direction and the range of the possible Poisson’s ratio is modeled statistically.

The resulting sensitivity coefficient is subsequently multiplied to the measurement reading ($MR_{x}$). The bridge factor *n* is respected according to the applied Wheatstone bridge circuit.

**Measurement amplifier**

Error arising from voltage metering is considered by an additive term $X_{rec,x}$ to the measurement reading ($MR_{x}$) using a Gaussian distribution of zero mean value and standard deviation equal to 13 of the maximum permissible error. Zero drift is only respected for the loaded state as described in Section 3.4.

**Thermal output**

The unavoidable thermal output $\epsilon_{S,X}$ of the sensor is incorporated by an additive to the measurement reading for both states and depends on the temperature difference ΔT to the reference temperature as described in Section 3.5. This variable comprises scatter in the apparent strain gradient and the correction factor for the coefficient of expansion of the concrete surface. To keep the property of unbiasedness, the mean value of the apparent strain results is specified in a previous run and, afterwards, subtracted from each strain value in the main simulation. The evaluated output for each load state no. i for strain measurement consists of the difference of the value from loaded state $\epsilon_{i}$ and reference measurement $\epsilon_{ref}$ as shown in Figure 11.

**Evaluation of strain**

In order to evaluate the divergence in the measurement results due to uncertainty in the involved parameters, the readings in the reference state $MR_{ref}$ and the loaded state $MR_{i}$ are computed in each iteration and subtracted to simulate the differential measurement.

**Simulation**

To simulate the density function of the possible readings, the evaluation is implemented in a Monte Carlo framework that repeats the evaluation of strain a large number of times using random samples from the distribution functions described in Section 3.1. The probability of the different parameters is assumed to be independent as this is appropriate in most cases, facilitates the evaluation, and trends to results in an estimation on the safe side. The check for a sufficient number of samples might be executed by statistical resampling (bootstrapping) as described in Section 2.2.2.

A subsequent sensitivity analysis, using global sensitivity measures, for example, is recommended to see the main influence factors and interactions of parameters in the model function.

## 5. Results of the Uncertainty Propagation

### Monte Carlo Study for Uncertainty Propagation

**Figure 12:**
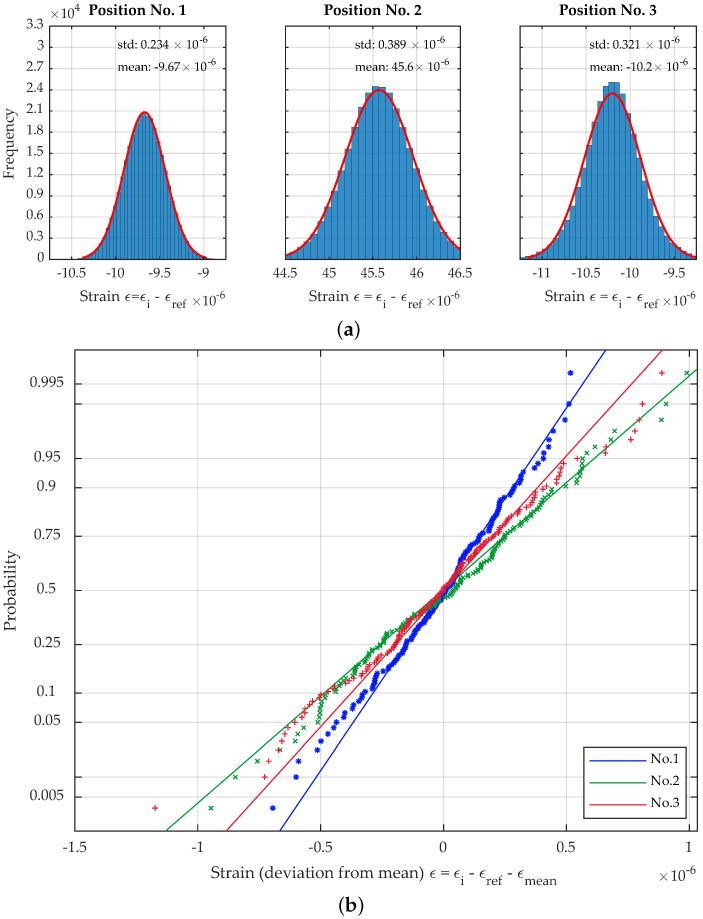
(**a**) Histogramms and fitted Gaussian PDF for the uncertainty propagation at position nos. 1–3. (**b**) Probability plot for the uncertainty propagation at position nos. 1–3 (every 2000th sample displayed).

The propagation of the measurement uncertainties through the model from Section 4 results in the histograms from Figure 12a. For each simulation, 300,000 samples were applied using Latin hypercube sampling for variance reduction. The calculation time for the simulation on an Intel(R) Xeon(R) Gold 6132 CPU @2.60 GHz was 24 s. The random samples were transformed using the inverse transform method to sample from the distribution functions described in Section 3.1. The different parameters were assumed to be mutually independent.

As the fit of the Gaussian PDF of the data shows, the results for the three load positions can approximately be described by a normal distribution. However, the accuracy of the fit is decreasing in the order of measurement. This observation is supported by the normal probability plot in Figure 12b and can be attributed to the increasing influence of temperature on the combined uncertainty due to its modeling as a boxcar function. The 95% confidence bounds for the mean and the standard deviation in Table 3 show that the applied number of simulations is by far sufficient and may be assumed much lower to speed up simulation for practical application.

## 6. Evaluation of Sensitivities

In order to reveal crucial contributions to the combined uncertainty in the results, a sensitivity analysis can give a starting point for the optimization of the measurement setup and interactions between the different factors. The Monte Carlo sample for calculating Sobol indices according to Section 2.3 was generated using 300,000 samples for $A,B,\;\mathrm{and}\;\mathrm{each}\;A_{B}^{\left(i\right)}$. The calculation time for the simulation on an Intel(R) Xeon(R) Gold 6132 CPU @2.60 GHz was around 7 s per evaluated measurement reading.

**Figure 13:**
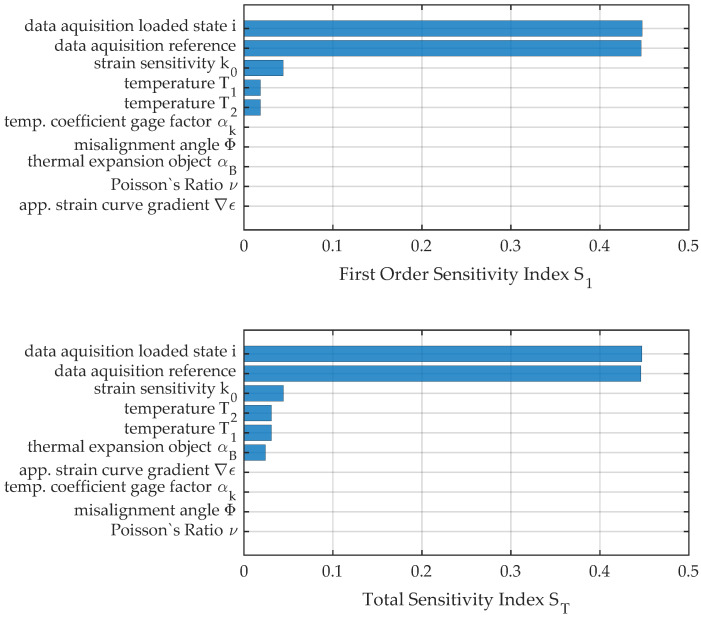
First-order and total sensitivity indices for load position no. 1.

The results in Figure 13 for the first load position and at a moderate temperature difference between the reference state of only 0.0515 K show that the sensitivity estimators of first order and the total indices approximately match and reveal a high influence of the data acquisition system in this stage of the test series. The resulting uncertainty can mainly be traced to the nonlinear behavior of the voltage measurement. The sensitivity measures $S_{1}\;\mathrm{and}\;S_{T}$ for temperature uncertainty are increased approximately by a factor of 1.5 but are still of minor influence. A more significant increase from the two measures of sensitivity can be seen for the importance of the thermal expansion coefficient $\alpha_{B}$. The increase by a factor of approximately 8 can be attributed to the interaction with temperature uncertainty in the model function of Section 4.

**Figure 14:**
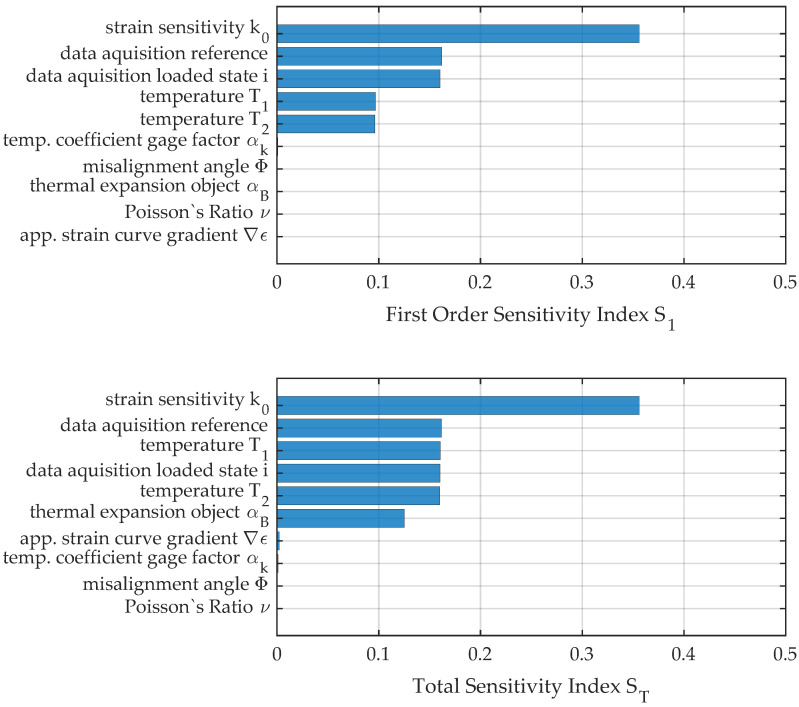
First-order and total sensitivity indices for load position no. 2.

The comparison between the two measures for load position no. 2 in Figure 14 reveals the influence of interaction terms for the quantification of uncertainty and sensitivity analysis.

**Figure 15:**
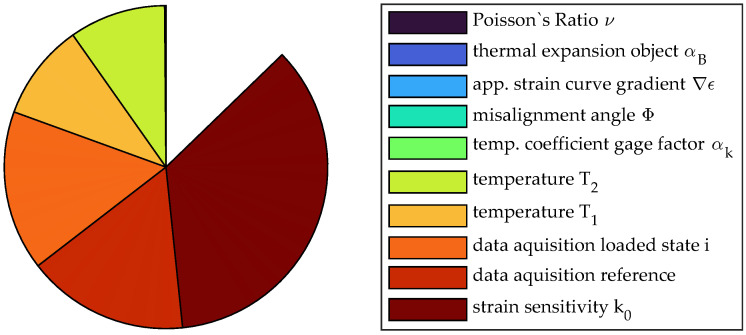
Pie chart of the sensitivity analysis of measurement no. 2 using sensitivity indices of first order.

Due to the significantly higher strain for position no. 2, the deviation of the strain sensitivity is of major importance while the influence of the data acquisition system has shrunk. Instead, the interaction effect from temperature prevails that would not have been seen if only sensitivity analysis or uncertainty propagation of first order was conducted. The increased temperature range of 0.195 K considered for the evaluation results in a significant influence of temperature and uncertainty in the thermal expansion of the measurement object. The considerable influence of the nonlinearity of the functional relationship f(x) and the importance for consideration in uncertainty evaluation can be seen in the pie chart in Figure 15. Only about 87% of the uncertainty can be explained by pure deviation of single factors, while 13% is attributed to interaction terms.

**Figure 16:**
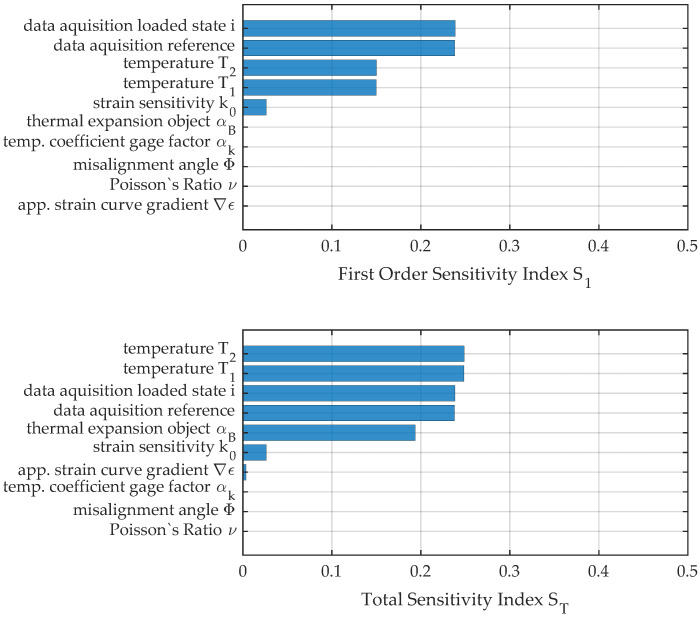
First-order and total sensitivity indices for load position no. 3.

**Figure 17:**
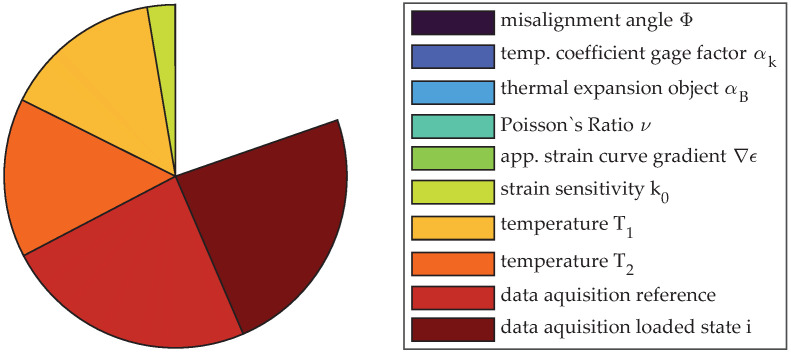
Pie chart of the sensitivity analysis of measurement no. 3 using sensitivity indices of first order.

Even worse than for measurement no. 2 is the effect of interaction between variables for load position no. 3, where about 83% of the resulting variance can be explained by first-order effects. Figure 16 gives the measures of sensitivity $S_{1}\;\mathrm{and}\;S_{T}$, and the pie chart in Figure 17 reveals the importance of interaction terms visually. Uncertainty due to change in temperature and especially its interaction with the thermal expansion coefficient of the measurement object $\alpha_{B}$ become the major reason for uncertainty. Additionally, the figures reveal the importance of incorporating the thermal expansion $\alpha_{B}$ into the propagation due to its interaction with variation of temperature. Those terms, depending on the absolute value of the measurement reading, however, decrease in importance for the chosen test procedure.

For all the different levels of strain and within the entire range of considered temperature, contributions from the gradient of the apparent strain curve, influence from temperature to the strain sensitivity of the gage, Poisson’s ratio of concrete, and the impreciseness of the application concerning angular misalignment have no considerable influence.

## 7. Conclusions and Outlook

This paper deals with several aspects of measurement uncertainty and simulation for strain measurement using electric strain gages. The main contribution to the scientific discourse can be summarized as follows:

- An experimental study on the expectable misalignment of the gage was conducted, and a probability model for the angle variation in the application process was derived.
- Based on physical relations, expertise in metrology, and engineering estimation, an uncertainty model is developed that comprises the entire measuring chain, but is sufficiently simple to be used in field application and practical engineering by inexperienced users.
- The advantages of Monte Carlo simulation over simple Gaussian error propagation are discussed in terms of correctness and applicability.
- In order to study the presented model for the uncertainty of strain measurement, sensitivity indices according to Sobol are introduced to measure the importance of different contributions to the combined uncertainty at different levels of strain and for different scatter in the external conditions (change in temperature).
- The importance of considering interactions due to the nonlinearity of the model function f(x) is studied. The results highlight the importance of the thermal expansion characteristics of the measured object by its high activeness in the interaction with temperature uncertainty.
- The presented workflow can serve as a template for the evaluation of uncertainties according to [10] and as a starting point for improving the accuracy of measurement. Sensitivity analysis via Monte Carlo simulation serves as a means to identify the driving factors behind the measurement uncertainty and those that can presumably be disregarded in further considerations. From this, conclusions about similar measurement projects can be drawn concerning the recommendable measurement equipment, the order of tests, and zeroing intervals.

Due to brevity and the restricted scope, not all questions arising could be tackled in this paper. Further studies may be conducted on the following:

- The application to different measurement materials and test setups;
- Possibilities to further simplify the implemented model based on the conducted sensitivity study;
- Integration of different assumptions in the temperature range, e.g., after improvement on the knowledge due to additional measurements;
- Extension of the presented model to include temperature hysteresis and mechanical hysteresis;
- The statistical modeling and the importance of bonding effects;
- Advancements concerning long-term measurements, e.g., in SHM or IoT applications;
- Implementation as a commercial software package.

## Figures and Tables

**Table 1:** Components of the assumable maximum error depending on the measurement reading (MR) and the supply voltage (SV) from the manufacturer’s specifications for DS-NET BR8 and the maximum value for nonlinearity according to the calibration certificate.

Uncertainty Component	Maximum Error of Component
Temperature	$\left[0.2\frac{\mu V}{V}\cdot SV+0.05\%\cdot MR\cdot U_{0}\right]\cdot \frac{\Delta T_{board}}{10\;K}$
Long-term drifting	$0.2\frac{\mu V}{V}\cdot \frac{\Delta T_{board}}{24\;h}\cdot SV$
Nonlinearity	$5\times 10^{-4}V$

**Table 2:** Probability distributions for examined parameters.

Random Variable	Probability Distribution	Reference
data acquisition system	$X_{Rec}∼N\left(0\;;\;\frac{{\left(\frac{\Delta U}{SV}\right)}_{i,max}}{3}\right)$	13 of maximum error from calibration data
gradient of the thermal output curve	$X_{\nabla \epsilon_{s}}∼U\left(1.0\frac{\mu m\;{}^{\circ}C}{m};1.8\frac{\mu m\;{}^{\circ}C}{m}\right)$	manufacturer’s specification
thermal expansion of concrete	$X_{\alpha_{B}}∼U\left(7\times 10^{-6};13\times 10^{-6}\right)$	[53]
strain sensitivity $k_{0}$	$X_{k_{0}}∼N\left(2.13;1.09\times 10^{-2}\right)$	manufacturer’s specification
angle of misalignment	$X_{\Phi }∼N\left(0.262{}^{\circ};0.422{}^{\circ}\right)$	experimental estimation
temp coefficient gage factor $\alpha_{K}$	$X_{\alpha_{K}}∼U\left(1.0\times 10^{-4}\frac{1}{K};2.0\times 10^{-4}\frac{1}{K}\right)$	manufacturer’s specification
Poisson’s ration of concrete	$X_{\nu }∼U\left(0.14;0.26\right)$	[52]

**Table 3:** Bounds of the 95% confidence intervals of the estimators for the mean and the standard deviation evaluated by bootstrapping using 10,000 resamples.

Measurement	Standard Deviation	Mean Value
No. 1	[0.2338;0.2501]	[−9.6732;−9.6716]
No. 2	[0.3884;0.3905]	[45.5700;45.5720]
No. 3	[0.3201;0.3219]	[−10.2030;−10.0200]
